# Review: Multimodal bioactive material approaches for wound healing

**DOI:** 10.1063/1.5026773

**Published:** 2018-06-26

**Authors:** Serena Mandla, Locke Davenport Huyer, Milica Radisic

**Affiliations:** 1 Institute of Biomaterials and Biomedical Engineering, University of Toronto, Toronto, Ontario M5S 3G9, Canada; 2 Department of Chemical Engineering and Applied Chemistry, University of Toronto, Toronto, Ontario M5S 3E5, Canada; 3 Toronto General Research Institute, University Health Network, Toronto, Ontario M5S 1L7, Canada

## Abstract

Wound healing is a highly complex process of tissue repair that relies on the synergistic effect of a number of different cells, cytokines, enzymes, and growth factors. A deregulation in this process can lead to the formation of a non-healing chronic ulcer. Current treatment options, such as collagen wound dressings, are unable to meet the demand set by the wound environment. Therefore, a multifaceted bioactive dressing is needed to elicit a targeted affect. Wound healing strategies seek to develop a targeted effect through the delivery of a bioactive molecule to the wound by a hydrogel or a polymeric scaffold. This review examines current biomaterial and small molecule-based approaches that seek to develop a bioactive material for targeted wound therapy and accepted wound healing models for testing material efficacy.

## INTRODUCTION

Delayed wound healing and chronic ulcers offer challenging complications to the healthcare system and are expected to affect 5.7 million Americans per year.^1^ More specifically, chronic foot ulcers are expected to affect 15% of people diagnosed with diabetes, a multifaceted disease in which the wound healing process is perturbed.^2^ Diabetic ulcer management is a leading cause of hospitalization, and in fact, diabetic ulcers are the underlying cause in about 85% of all diabetic limb amputations.^3^ Treatment of diabetic foot ulcers is estimated to cost US$20 billion annually,^1^ thus highlighting the economic burden caused by non-healing chronic wounds. Therefore, it is evident that an optimal solution must combine and address multiple biological characteristics associated with wound healing (prolonged inflammation, matrix regeneration, poor angiogenesis, and re-epithelialization).^2^

**FIG. 1.**
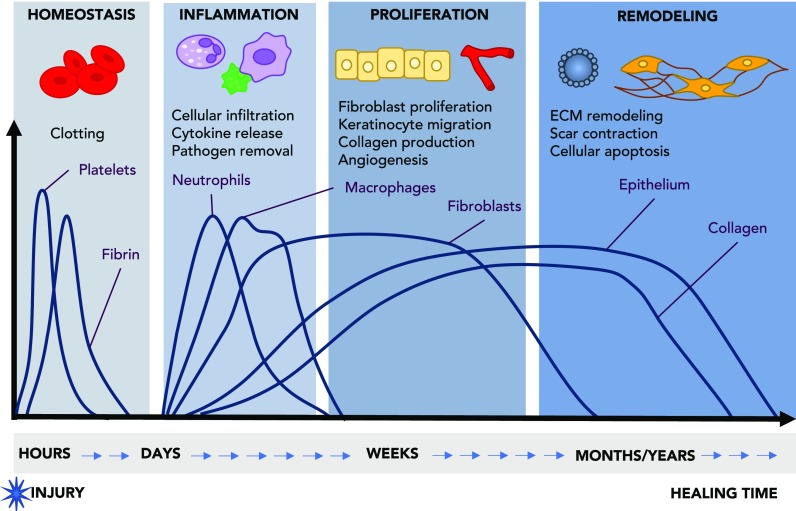
Overview of the timeline of interconnected phases of wound healing. Upon achieving homeostasis and clotting, the wound progresses through a period of inflammation, proliferation, and remodeling. During inflammation, immune cells infiltrate the wound bed to remove pathogens and release cytokines which lead to downstream signaling necessary for the recruitment of other cells and production of ECM. Following inflammation which typically lasts a couple of days, fibroblasts are recruited and begin producing collagen. This stage is also hallmarked by re-epithelialization and angiogenesis. Finally, the wound enters the final stage of remodeling in which there is scar contraction, ECM remodeling, and cellular apoptosis. Adapted with permission from Shechter *et al.*, Trends Mol. Med. **19**(3), 135 (2013). Copyright 2013 Elsevier.^10^

Human skin is composed of two distinct layers, namely, the epidermis and the dermis. The epidermis is the outer most layer and it is primarily composed of keratinocytes, followed by melanocytes, Langerhans cells, and Merkle cells. The dermis is composed of extracellular matrix (ECM) components such as collagen, elastin, and glycosaminoglycans, with fibroblasts being the primary cell type. The dermis is highly vascularized and is also home to dermal adipose cells, mast cells, and infiltrating leukocytes.^4,5^ The skin acts as the primary defense between the body and the environment.^5^ Upon injury, it is imperative that the wound heals quickly and effectively to prevent exposure to pathogens and chemical insults. The process of wound healing is a complex progression of various biological and cellular cues and can be broken down into 3 stages: inflammation, proliferation, and remodeling (Fig. 1).^6–10^ Immediately following tissue damage, the clotting cascade is activated, and hemostasis is achieved through the formation of a clot formed by collagen, fibrin, and platelets. The fibrin clot serves as a preliminary scaffold for infiltrating cells, and the clotting cascade continues to release a number of growth factors that activate the immune system.^7^ Neutrophils are the first cell recruited to the wound and are responsible for removing any pathogens.^8^ Within 2–3 days, monocytes appear and differentiate into macrophages, releasing several pro-inflammatory cytokines responsible for coordinating host defense, removal of apoptotic cells, cell proliferation, and tissue and ECM remodeling.^11^

The second stage of wound healing can be characterized by new tissue formation and cellular proliferation and typically lasts between 2 and 10 days. The epidermis, or the outermost layer of the skin, is largely composed of keratinocytes. These cells are responsible for migrating into the wound upon injury in a process known as re-epithelialization. Formation of new blood vessels follows, in which new capillary sprouts form, and fibroblasts and macrophages replace the fibrin clot with granulation tissue. This new tissue promotes the migration and proliferation of keratinocytes at the leading edge of the wound, and endothelial cells to form new blood vessels.^7^ Finally, some fibroblasts can be stimulated by macrophages to differentiate into myofibroblasts, which are contractile cells responsible for closing the wound. Together with myofibroblasts, large amounts of collagen are produced, which may form the scar.^8^

Remodeling is the final stage of wound healing and typically begins two weeks after injury, and this can last up to a year depending on the healing characteristics. During this phase, the deposited collagen and matrix are reorganized by fibroblasts, macrophages, and endothelial cells. Following remodeling, the remaining cells either undergo apoptosis or recede from the wound bed.^8^ Unfortunately, the complete reorganization of the collagen to resemble healthy tissue is not often possible, as evidenced by the remaining scar on the skin.^7^

When the wound healing process progresses as expected, the final result is intact tissue with functional resemblance to healthy tissue; however, full wound healing is hindered if there are pathological deficiencies within the healing process. An over-proliferation and non-migratory keratinocytes can lead to epidermis thickening and keloid formation at the wound edge.^2,6^ Another hallmark of non-healing wounds is the extended presence of neutrophils or macrophages, which can result in a deregulation of pro-inflammatory cytokines. Downstream consequences include degradation of the ECM, inhibition of cellular migration, and the release of excess reactive oxygen species (ROS), resulting in tissue damage.^2,9,11^ Delayed wound healing in diabetics is largely thought to be attributed to non-migratory and over-proliferative keratinocytes, as well as a prolonged inflammatory response leading to excess ROS.^2,6^ Furthermore, diabetic chronic wounds can be characterized by inadequate angiogenesis resulting in a decreased blood flow to the wound bed.^12^

Standard wound care consists of debridement to remove non-viable tissue and bacterial biofilms, followed by wound dressing and offloading therapy in the case of a foot ulcer.^6^ Popular wound dressings consist of standard cotton gauze, highly absorbent dressings, such as collagen and alginate, or hydrocolloids.^6,13^ However, these basic tenets of wound care are often ineffective at treating the demands of chronic wounds, causing healthcare professionals to seek advanced therapies. One of the first Food and Drug Administration (FDA) drugs aimed at accelerating wound healing is the chemical entity, Regranex, which consists of a recombinant human platelet-derived growth factor (rhPDGF) in a gel. However, the FDA issued a warning regarding the use of Regranex, as it has been linked to increased risks of metastatic cancer.^14^ Therefore, the challenge lies in creating a multifaceted dressing which has the capability to positively affect most wound types. This can only be achieved through a multi-dimensional approach which utilizes bioactive additives for a targeted effect. This review serves to outline some of the current approaches which are aimed at targeting a specific deficiency within the wound healing niche.

## GROWTH FACTOR DELIVERY AND MICROENVIRONMENT RECAPITULATION

Growth factor delivery to chronic wounds presents an advantage in its ability to directly deliver growth factors to an area in deficit, in hopes of triggering a coordinated cascade of cellular responses.^13,15,16^ Growth factors have a role in cellular migration, proliferation, and adhesion.^17^ Target cells include keratinocytes and fibroblasts, which are involved in re-epithelialization and collagen deposition, respectively.^17^ Currently, there exists one FDA approved growth factor loaded gel, Regranex, which contains 0.01% rhPDGF. Early clinical trials revealed increased granulation tissue and re-epithelialization in rabbit ear excisional models and a significant increase when human patients with diabetic foot ulcers were treated.^3,18^ However, another study was unable to reproduce the accelerated wound healing results of rhPDGF in their diabetic mouse model, despite observing increased granulation tissue,^19^ and repeated exposure has been linked to increased cancer malignancy by the FDA.^16^ Regranex gel has defined our understanding of growth factor mediated gels for wound healing, and the use of PDGF has been common for many years.^20–24^ Other commonly used growth factors include the epidermal growth factor (EGF),^25–31^ fibroblast growth factor (FGF),^21,26,27,32,33^ and vascular endothelial growth factor (VEGF).^34–36^ Table I has a summary of growth factor modified materials and their corresponding strategies for growth factor encapsulation and delivery.

**TABLE I.** Summary of growth factor biomaterials and the method of the drug encapsulation and delivery. PDGF, platelet derived growth factor; FGF, fibroblast growth factor; EGF, epidermal growth factor; VEGF, vascular endothelial growth factor; and PRP, platelet rich plasma.

Growth factor	Delivery system	Method	*In vivo* wound model	References
PDGF	Regranex (sodium carboxymethylcellulose gel)	Mixing	Diabetic patients with non-healing, full thickness diabetic foot ulcers	23
PDGF	Chitosan gel	Mixing	Rats–streptozotocin induced diabetes (full thickness wound)	24
PDGF	Collagen-chitosan gel	Mixing	Rats (dorsal wound)	22
PDGF or FGF	Polyethylene glycol	Mixing	Db/db mice (full thickness wound)	21
FGF-2	Heparin-poly(ethylene argininylaspartate digylceride) matrix	Coacervation	Mice (full excisional wound)	33
FGF	Polyurethane hydrogel	Mixing	Mice and rats (full excisional wound)	32
EGF or FGF	Hyaluronate-collagen dressing	Mixing, lyophilization	Mice–streptozotocin induced diabetes (full thickness wound)	26 and 27
EGF	Polyvinyl alcohol-alginate gel	Mixing, cross-linking	Rats–streptozotocin induced diabetes (full thickness wound)	28
EGF	Pluronic-chitosan gel	Mixing, cross-linking	Mice–streptozotocin induced diabetes (dorsal burn)	25
EGF	Hyaluronate	Aldehyde-amine conjugation	Rats (full excisional wound)	30
EGF	Poly-lactic-*co*-glycolic acid	Electrospinning nanofibers	Db/db mice (full thickness wound)	31
VEGF DNA	Chitosan scaffolds	Freeze dried chitosan soaked in DNA solution	Rats (full excisional wound)	35
VEGF	Alginate microspheres	Microencapsulation, ion exchange	Rat (incision in groin)	36
PRP	Platelet gel	Thrombin and calcium activation	Diabetic patients with non-healing foot ulcers	44

Common strategies for delivering growth factors is mixing and encapsulating the growth factor in a biomaterial delivery system^16^ (Table I). This is the simplest strategy, and results in a rapid release of the growth factor followed by a slow sustained release.^32^ This release profile allows for a rapid burst delivery of the growth factor; however, there are other encapsulation strategies better suited to sustained delivery. Wu *et al.* developed a heparin-based coacervate using FGF-2 as the cargo, and poly(ethylene argininylaspartate digylceride) as the matrix. The FGF2 coacervate had a prolonged release, with only 60% of the growth factor being released by 17 days, which can support long-term delivery of the growth factor to the wound environment.^33^ Alternatively, the growth factor can be chemically conjugated to the delivery material hydrogel. Kim *et al.* conjugated the amine terminal of EGF to an aldehyde modified hyaluronate. The EGF was released relatively slower in comparison to the non-conjugated EGF; however, nearly 90% of the EGF was released by 8 h. Nevertheless, this approach extended the bioactivity of the conjugated EGF in comparison to EGF alone, which is an advantage as growth factors tend to have a short half-life.^30^

This field, however, has faced challenges derived from the low stability of growth factors in an *in vivo* setting, a short half-life, and unwanted side effects as a result of immunogenicity of recombinant proteins.^16,37^ As an alternative, growth factor gene delivery has seen an increase in popularity for its long shelf life and stability, in conjunction with low cost for manufacturing. The use of materials containing complementary DNA (cDNA) has led to an extended period of high growth factor concentration with more recognizable proteins in comparison to recombinant proteins.^15^ Building off the success of recombinant PDGF, cDNA encoding for PDGF has been loaded into collagen gels for wound healing applications. Early experiments observed an increase in granulation tissue and proliferating cells in the wound bed of a rabbit model.^38,39^ PDGF encoding cDNA has since been used in human trials and has seen early success in accelerating would closure in diabetic foot ulcers.^40,41^

An alternative to the external source of growth factors is to harvest a natural source of growth factors from platelet-rich-plasma (PRP) from the patient themselves or a healthy donor.^37,42^ PRP is a rich source of growth factors and platelets which assist with clot formation and serve as a positive feedback for the release of a number of other factors necessary for wound healing.^43^ PRP gel is produced using patient blood which has been centrifuged to separate the red blood cells from the PRP. Thrombin is then added to the PRP to create a bioactive gel which can be applied to a wound.^44^ In an equine wound model, wounds treated with a PRP gel exhibited a multifaceted healing response to the PRP gel. Treated wounds had an increase in re-epithelialization, keratinocyte differentiation, and collagen alignment, suggesting the therapeutic benefit of PRP for its high concentration of growth factors necessary to stimulate healing.^43^ In a randomized and controlled clinical trial, patients with diabetic foot ulcers of at least 4 weeks were treated with a PRP gel or a normal saline gel weekly until wound closure. Wounds treated with the PRP gel were significantly more likely to heal in comparison to the normal saline gel, and the treatment was well tolerated by patients, resulting in minimal adverse events.^44^

## RE-EPITHELIALIZATION

**FIG. 2.**
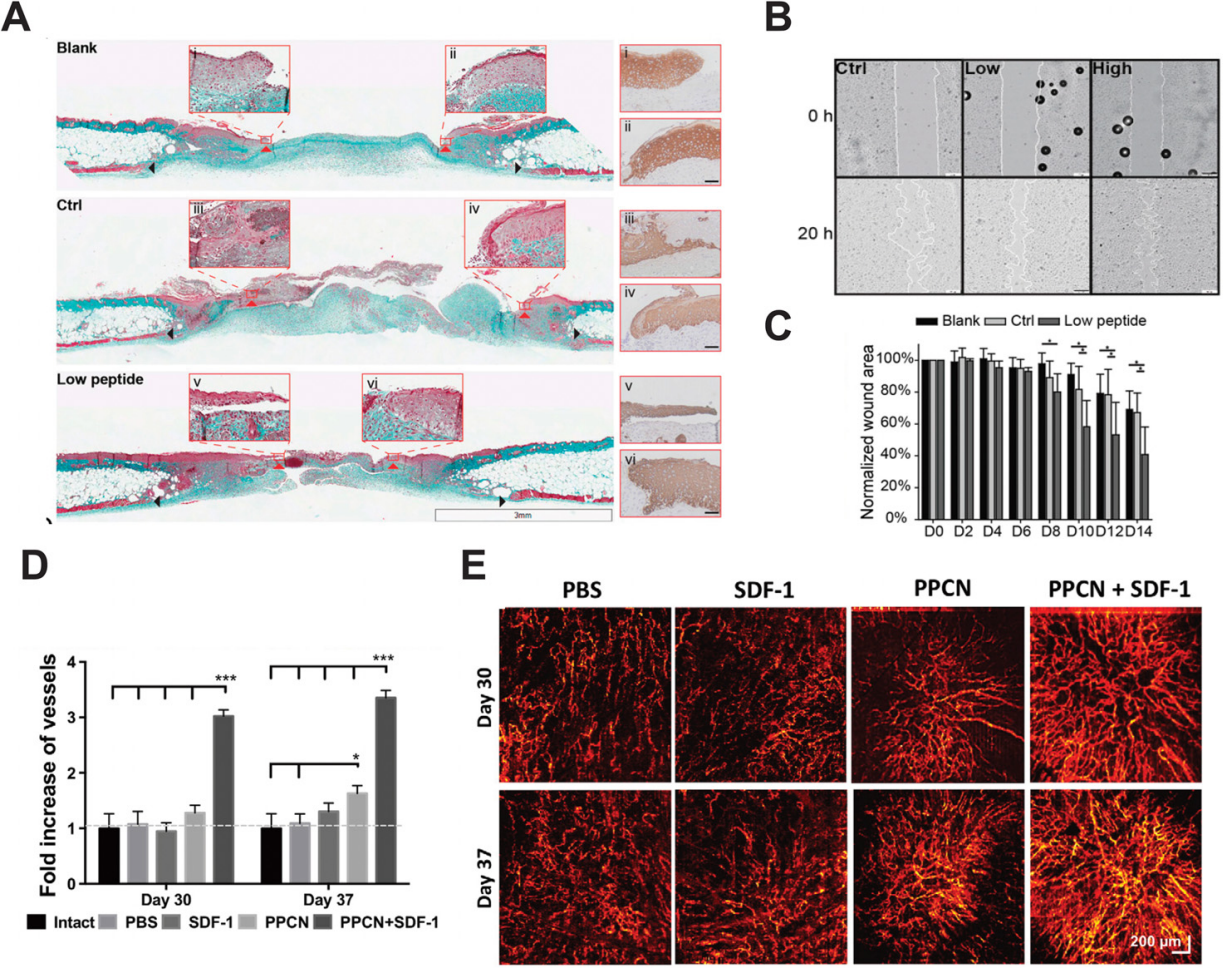
Advanced wound therapies aimed at accelerating wound closure by increasing the rate of re-epithelialization and angiogenesis. (a)–(d) Angiopoietin-1 derived peptide, QHREDGS, immobilized to a collagen-chitosan hydrogel. (a) Masson's trichrome stained cross sections of wounds in diabetic mice treated with no hydrogel, peptide free hydrogel, and QHREDGS conjugated hydrogel 14 days after wounding. Red arrowheads indicate the tips of the epithelial tongue, and black arrowheads indicate wounds edge. (Scale bar = 3 mm) Insets have been stained with pan-keratin to confirm epithelial tongue. (Scale bar = 50 *μ*m). (b) Human epithelial keratinocytes seeded on no peptide control film, and films containing low (100 *μ*M) and high (650 *μ*M) concentration of QHREDGS peptides indicate that peptide containing films accelerate keratinocyte migration. (c) Quantification of the wound size reveals faster wound closure in wounds treated with the peptide hydrogel. Reproduced with permission from Xiao *et al.*, Proc. Natl. Acad. Sci. U. S. A. **113**(40), E5792 (2016). Copyright 2016 National Academy of Sciences.^2^ (d)–(e) SDF-1 entrapped in PPCN. (d) Quantification of blood vessel density following wound treatment with the PBS control, SDF-1 alone, PPCN alone, and SDF-1 entrapped in PPCN reveals a significant increase in vessel density. (e) Microangiography of the skin following treatment and healing on days 30 and 37. Reproduced with permission from *Zhu *et al**., J. Controlled Release **238**, 114 (2016). Copyright 2016 Elsevier.^53^

In order for full wound closure to occur, keratinocytes, or the major cell type in the epidermis, must migrate across the wound bed to close the wound surface. In non-healing wounds, keratinocytes are non-migratory and effective re-epithelialization cannot commence.^45^ Keratinocyte migration is a complex process, depending on a variety of factors capable of orchestrating the dissolution of cell-cell and cell-ECM interactions, and the upregulation of factors necessary for migration.^45^ Current mechanisms of action consist of increasing granulation tissue formation to provide a substrate for keratinocyte migration, and supplying growth factors and cytokines to attract keratinocyte migration.^45–47^ However, conventional growth factors, and PDGF for that matter, are limited in their ability to interact with keratinocytes as keratinocytes lack PDGF receptors.^48^ Xiao *et al.* created a novel hydrogel consisting of an angiopoietin-1 derived peptide, QHREDGS (glutamine-histidine-arginine-glutamic acid-aspartic acid-glycine-serine), immobilized to a collagen-chitosan hydrogel. The functionalized hydrogel promoted keratinocyte migration *in vitro* [Fig. 2(b)] and was shown to protect against harmful reactive oxygen species, which are frequently generated in chronic wounds. When applied as a treatment on an excisional wound on a diabetic mouse model, the peptide hydrogel significantly accelerated re-epithelialization and closure of the wound, while increasing granulation tissue formation [Figs. 2(a) and 2(c)].^2^ The QHREDGS peptide interacts with cells though β_1_-containing integrins. Keratinocytes express the α_3_β_1_ integrin which implicates its survival and migration.^2^ In a similar approach, a group led by Li encapsulated a small fragment of the secreted Hsp90α in carboxymethylcellulose. The Hsp90α gel was shown to increase motility in human keratinocytes, human dermal fibroblasts, and human dermal microvascular endothelial cells, under both healthy conditions and hyperglycemic conditions.^49^ Full excisional wounds on a diabetic pig treated with the Hsp90α gel indicated increased re-epithelialization and granulation tissue deposition in comparison to control wounds treated with the industry standard Regranex.^48^ Similarly, keratinocytes and dermal cells express the LRP-1 receptor which is implicated in cell motility and Hsp90α signaling.^49^ Cell motility is largely driven through integrin signaling; therefore, it is necessary to find additional molecules which can promote re-epithelialization by targeting integrins involved in cell motility.^49^

## ANGIOGENESIS

Angiogenesis is a critical event in the progression of wound healing. Disruption of this process can impede tissue formation and is commonly observed in diabetic wounds.^50^ VEGF plays a major role in promoting angiogenesis and is common in wound dressings aimed at increasing vascularization. A phase 1 trial was aimed at assessing the safety and efficacy of recombinant human VEGF which was topically applied to chronic diabetic ulcers. Preliminary results demonstrate that the VEGF was well tolerated;^51^ however, growth factor delivery is hampered by the short half-life of the growth factors, and VEGF has been linked to the creation of leaky vasculature and fenestrations.^52^ As such, alternatives to growth factor delivery have been explored for targeting angiogenesis. Lord *et al.* constructed a perlecan-VEGF transgene and loaded it into a chitosan scaffold. The scaffold was able to successfully transfect keratinocytes *in vitro* and upregulate the expression of perlecan and VEGF in treated rat wounds, leading to more vessel formation and improved tissue maturation.^35^ In a similar study by Zhu *et al.* aimed at stimulating angiogenesis, stromal cell derived factor-1 (SDF-1) was entrapped in poly(polyethylene glycol citrate-*co*-N-isopropylacrylamide) (PPCN). In a diabetic mouse model, wounds treated with the PPCN + SDF-1 had a significantly denser perfusable vascular network upon wound closure in comparison to the control, SDF-1, and PPCN alone, suggesting a synergistic effect when SDF-1 is slowly released from the PPCN over an extended period of time [Figs. 2(d) and 2(e)].^53^ Other approaches which aim at upregulating the expression of VEGF include a curcumin and pluronic gel.^54^

## MATRIX METALLOPROTEINASE INHIBITION

**FIG. 3.**
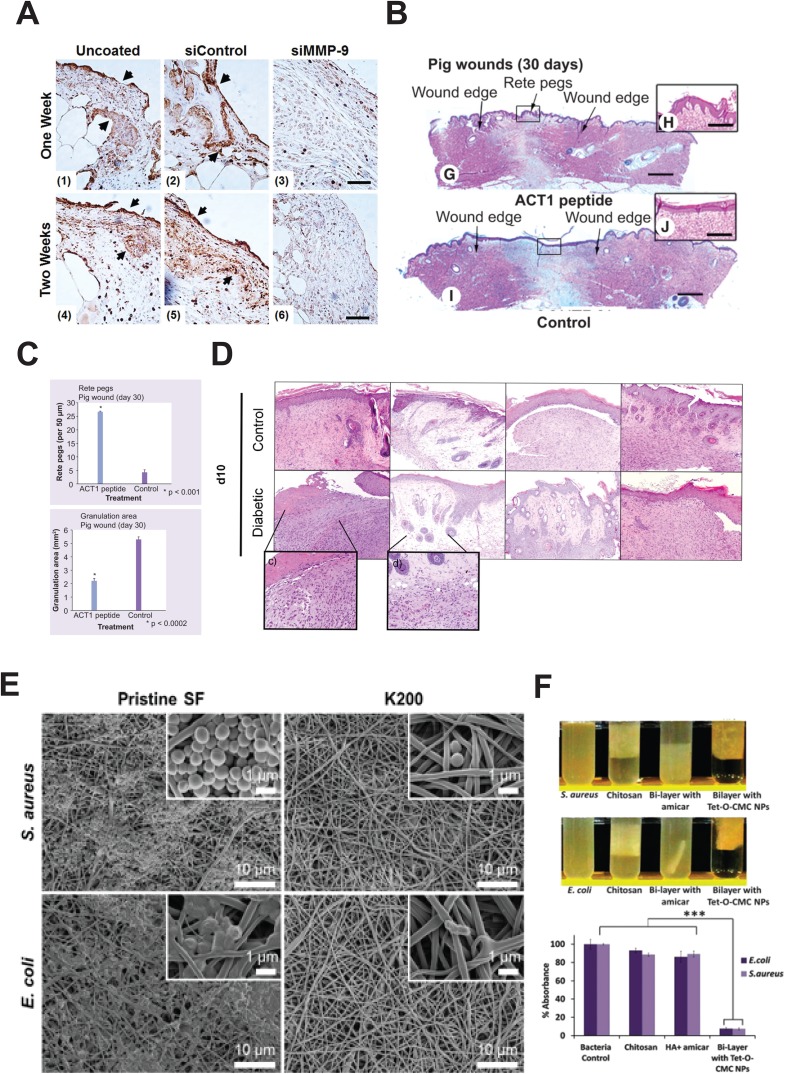
Wound therapies targeted at treating the demands of chronic wounds which impede the progression of wound healing through the classical phases. (a) RNA interference using small interfering RNA targeted to silence MMP-9 was self-assembled into a hydrolytically degradable wound bandage. Immunohistochemistry reveals that wounds treated with the siMMP-9 show less staining for MMP9 in comparison to the uncoated control, and the siControl bandage. Black arrowheads indicate regions with increased staining. Reproduced with permission from Castleberry *et al.*, Adv. Mater. **28**(9), 1809 (2015). Copyright 2015 John Wiley & Sons.^55^ (b) and (c) Connexin 43 peptide dissolved in a pluronic gel reveals recovery of physiological features in the wounded tissue. (b) H&E histology of wounds treated with pluronic gel with and without the connexin 43 peptide on day 30 reveals increased return of rete ridges in the connexin 43 peptide treated group. (c) Quantification of rete ridges and granulation tissue shows a significant increase in rete ridges and decrease in granulation tissue in the healed tissue in a pig wound healing model Reproduced with permissions from Gourdie *et al.*, Regen Med. **4**(2), 205 (2009). Copyright 2009 Future Medicine.^61^ (d) H&E staining of wounds treated with a no treatment control, hydrogel alone, neurotensin alone, and neurotensin impregnated hydrogel reveals fewer inflammatory cells in the neurotensin hydrogel suggesting modulation of the immune system. Reproduced with permissions from Moura *et al.*, Acta Biomater. **10**(2), 843 (2014). Copyright 2014 Elsevier.^68^ (e) and (f) Antimicrobial strategies in wound healing treatments. (e) SEM images of silk fibroin scaffolds immobilized with antimicrobial Cys-KR12 peptide show limited bacterial growth when compared to pristine controls. Reproduced with permissions from Song *et al.*, Acta Biomater. **39**, 146 (2016). Copyright 2016 Elsevier.^85^ (f) Encapsulation of Tet-O-CMC nanoparticles in collagen sponges demonstrates *in vitro* efficacy against bacterial strains. Reproduced with permissions from Mohandas *et al.*, J. Mater. Chem., B **3**(28), 5795 (2015). Copyright 2015 Royal Society of Chemistry.^82^

While delivery of deficient growth factors to a wound site accelerates wound closure, their effectiveness is argued to be hindered as a result of proteolytic activity in the wound.^55^ As such, impregnating hydrogels and biomaterials with matrix metalloproteinase (MMP) inhibiting molecules or proteases is likely to positively affect wound healing by reducing degradation of growth factors within the wound. The primary mode of action for inhibiting MMPs is through the addition of a chelating agent, which binds to the active zinc sites on the MMPs, blocking its activity as a result. Some chelators within this field include 4-vinylbenzyl chloride chemically immobilized to atelocollagen,^56^ 2,3-dihydroxybenzoic acid conjugated to gelatin microspheres,^57^ and bisphosphonate loaded in a poly(2-hydroxy methacrylate).^58^ In an alternative approach to chelators, Castleberry *et al.* designed a bandage using a layer by layer approach in which poly(β-amino ester) 2 and dextran sulfate were alternately deposited onto a nylon surface. The bandage was then coated in chitosan and a MMP-9 small interfering RNA (siRNA) strand. The siRNA coated bandages increased the collagen content in the granulation tissue of treated wounds in mice and decreased MMP9 expression and activity in comparison to the uncoated control [Fig. 3(a)].^55^ The delivery system developed by Castleberry *et al.* could be extended to deliver a variation of siRNA for target wound treatment.

## SCAR MINIMIZATION

Formation of scars is an expected result of wound healing, especially in the case of non-healing chronic wounds.^59^ For many, scar formation is extremely undesirable and often of significant importance. As the wound heals, collagen is deposited to the wound bed. This new collagen typically has an unorganized structure, poor strength, and low flexibility, thus resulting in a visible scar on the surface of the skin.^59^ The gap junction protein connexin 43 (Cx43) has roles in wound healing, re-epithelialization, and ECM composition.^60^ A small Cx43 mimetic peptide immobilized in 20% pluronic acid produced more evenly distributed vasculature and epidermal rete ridges and less granulation tissue, suggestive of low scar formation [Figs. 3(b) and 3(c)].^61^ While the exact mechanism of action remains unknown, Cx43 has been known to a regulate transforming growth factor beta (TGF-β) signaling, a marker of fibrosis.^61^ Early human trials saw relative success of the material in reducing scar formation following surgery^62^ and healing diabetic foot ulcers.^63^ Phase 3 clinical trials are ongoing to evaluate the efficacy of this material in diabetic chronic foot ulcers.^64^

## IMMUNE MODULATING SMALL MOLECULE DELIVERY

Prolonged inflammatory phases are a hallmark of delayed wound healing. While inflammation is an important factor for successful wound healing, non-healing chronic wounds are often trapped in a prolonged state of inflammation.^2,6,65^ This prolonged inflammation is characterized by oxidative stress which leads to keratinocyte injury further delaying wound healing.^2^ In diabetic wounds specifically, there is an increased recruitment of neutrophils to the wound site by the keratinocytes,^66^ leading to a hostile wound environment, which is unable to progress through the stages of wound healing. Therefore, modulating the immune system and regulating the transition from the inflammation phase to proliferation phase are believed to be critical in wound closure.^67^ In an effort to modulate the immune response, Moura *et al.* loaded a chitosan sponge with neurotensin, a neuropeptide, which interacts with macrophages, leukocytes, dendritic cells, and mast cells. *In vivo* work on mice showed an immediate decrease in the wound size and overall improvement in wound quality in comparison to the controls, as well as a decrease in the tumor necrosis factor alpha (TNF-α) protein content, a hallmark for increased MMP9 production and inflammation [Fig. 3(d)].^68^ In a similar approach, the neuropeptide substance P has been shown to induce an acute inflammatory response at early time points, following by macrophage polarization to M2 or alternatively activated macrophages. It is proposed that activation of macrophages to an M2 phenotype and an acute inflammatory response enabled the progression of the wound to the proliferative phase, thus acting to reverse the chronic inflammation.^69^ However, substance P has a short half-life and is easily degraded. As a result, it was loaded into chitosan liposomes in an attempt to extend the half-life. In a proof-of-principle study, substance P loaded liposomes promoted keratinocyte migration in a wound healing assay and showed “programmable” peptide release, demonstrating the prospects of a substance P loaded liposome for wound healing.^70^

## ANTIBACTERIAL TREATMENT STRATEGIES

Bacterial load in a wound healing environment has a significant impact on the ability to achieve efficient closure. An infection occurs when a foreign microorganism competes with the host immune system, inciting a response in an effort to eliminate the unknown.^71^ Often this goes unnoticed, but pathogenic bacteria tend to provide especially difficult resolution.^72^ This has severe implications on the delay of wound healing and is often heightened by the generation of bacterial biofilms by organisms including *Staphylococcus aureus* and *Pseudomonas aeruginosa.*^73^ Given these concerns, significant efforts have been made in wound healing materials to minimize and treat wound infections.

The gold standard in commercially available infection fighting wound treatments relies on the antibacterial properties of silver.^74^ Silver has also been suggested to provide further benefits to the wound healing process, including the downregulation of MMPs.^71^ Effective in many forms, recent efforts have focused on the delivery of silver nanoparticles encapsulated in dressings for improved infection prevention.^75,76^ Using a chitosan-polyphosphate dressing, Ong *et al.* demonstrated effective antimicrobial and blood clotting properties as a supportive healing tool at the wound surface.^77^ Similar results were seen using a chitin material base to prevent chronic infection.^78^ A number of commercially available dressings rely on this technique in the clinic, where silver based treatments are embedded within wound dressings and treatments to achieve antibacterial efficacy.^79^ Although efficacy is strong, there are concerns surrounding silver toxicity to other cell types, and therefore interest in studying alternative treatments.

Similar approaches have been applied with antibiotic drugs as active agents, minimizing local infection through controlled release and localized treatment. This provides benefit over traditional systemic delivery, as drugs can be given in a concentrated dose local to the pathogen. As with silver, most research here focuses on improving delivery systems to better deliver drug payload.^80^ The use of a chitosan hydrogel system has become a common delivery method, owing to its favourable release properties and demonstrated inherent antimicrobial efficacy.^81^ In combination with a bi-layered intelligent scaffold design, a chitosan containing nanoparticles demonstrates bivalent efficacy in the delivery of anti-bacterial and pro clotting factors [Fig. 3(f)].^82^ Work by Pawar *et al.* loaded streptomycin and diclofenac into polymer film blend of PolyOx with hyprophilic polymers and demonstrated the tunability of release kinetics.^83^ Although achievement of localized delivery has been well demonstrated in the literature, the inability of antibiotic drugs to combat multi-resistant organisms has limited the continued efficacy of these approaches.

With rising concerns of antibiotic resistance to common treatments such as silver and antibiotic drugs, some have looked to utilize different methods for minimizing wound infection. In one approach, Zhou *et al.* instead focused on tailored release of antibacterial agents in response to the presence of pathogenic bacteria, coupled with a visual marker to indicate potential infection.^84^ In a different approach, Song and colleagues delivered a short chain antimicrobial peptide from an electrospun network, demonstrating effective antimicrobial activity against common strains [Fig. 3(e)].^85^ The utilization of peptide based therapies presents an attractive alternative against resistive bacteria.^86^ Other alternative treatments have also been explored, including the impact of honey on infection prevention.^87,88^ These strategies that look beyond the typical silver and antibiotic based treatments provide potential alternatives to combat antibiotic resistance.

## WOUND HEALING MODELS

In the thorough assessment of wound healing materials, potential compounds are subject to stepwise testing to determine efficacy. Most meaningful results tend to arise with testing *in vivo* for favorable outcomes including wound closure, infection reduction and prevention, and favorable phenotypic healing characteristics. With such assessments, the selection of an appropriate animal model is essential to adequately assess and collect relevant and meaningful results (Table II).^89^ The wound healing paradigm has been thoroughly investigated, with a range of available models according to the desired conditions of assessment.^90^

**TABLE II.** Comparison of prevalent animal species used as *in vivo* wound healing models.

Model	Advantages	Disadvantages
Rodent	• Strong understanding of genetic makeup	• Skin characteristics present differences in the healing mechanism
	• Multiple specialized models available	• Differences in hair growth
	• Chronic healing models available	• High physical activity of animal can impact wound healing environment
	• Relatively low cost to house and maintain	• Irrelevant wound size
	• Simple surgical operations	
Pig	• Skin is similar to humans	• Lack of specialized models
	• Comparable healing characteristics to humans	• Ethical considerations
	• Generation of wounds at relevant scales	• Complex surgical operations
		• High cost to house and maintain
		• Need for specialized facilities

Rodent based models have long been utilized as an initial step for *in vivo* wound healing models. These models provide ease of a small body size, well understood genetics, and predicable experimental design. The most basic model involves the generation of a skin wound on the back of the rodent of known diameter, allowing for observation of wound closure with application of the bioactive agent.^91^ Models can be adapted from this concept to various shapes and sizes dependent on the clinical analogue under study. This includes the generation of a round wound using a biopsy punch,^92^ differentiating dimensions with surgical scissors,^93^ or burn models generated with lasers, hot metal probes, or ultraviolet light.^94^ Wounds can be left open to heal or treated with splits, sutures, or specific dressing to mimic desired healing characteristics.^95^ In many cases, the wound is sealed with Tegaderm dressing, developing a “moist” healing environment.^96^ Given the flexibility of these models, results vary in the literature according to the initial wound formation and allow for differential treatment through systemic, localized, topical, or dressing dependent active agents. Further complexity can be introduced through the ease of genetic manipulation of these models, incorporating immunodeficiency,^97^ hairlessness,^98^ and diabetic characteristics,^99^ among others. This is often complemented by external challenge, such as bacterial infection, to establish models for chronic wounds.^99,100^

Although rodent models provide a number of advantages, their physiological differences from the healing characteristics of a human wound can impact the applicability in translation. Although species share over 85% genetic similarity,^101^ primary differences lie in their skin types, as rodents have loose skin attachment to the subcutis, whereas human skin is relatively tight.^102^ As a result, much of wound closure occurs as contraction in rodent models, whereas in the clinic a therapeutic agent may be better served to focus on the primary role of reepithelization as a treatment strategy for human wounds.^103^ This necessitates careful consideration when assessing experimental outcomes for clinical applications. Although there are differences between contraction conditions in rodent models, all animal models are limited as they cannot effectively mimic the clinical condition of non-healing wounds. This can lead to a discrepancy in clinical applicability when compared to research success.

In an effort to better mimic human wounds, researchers tend to next assess treatment strategies in a pig model. This tends to be a secondary animal model, limited by ethical and economic concerns of implantation.^94^ Although when considering pigs from a biological relevance perspective their wound characteristics demonstrate much greater similarity to a human wound healing. Skin contains similar lipid and protein makeup is firmly fixed to the subcutis, and heals primarily by re-epithelialization.^104^ Limited adoption could be attributed to the lack of transgenic models, limiting research to later stage product translation toward clinical application.^105^ As such, the research space could benefit from outsourcing of such experiments to facilities that can undertake such studies with greater ease.

## CONCLUSION

Wound healing is a complex process in which the tissue progresses through 3 highly regulated and distinct phases. Dysregulation or disruption of this process results in non-healing chronic wounds which are difficult to treat. The complex wound healing regime demands that an optimal biomaterial be able to address multiple biological wound healing characteristics. This review served to highlight recent multimodal, bioactive biomaterials which target a deficit within chronic wounds. The delivery of growth factors, peptides, RNA and cDNA, and antibiotics to chronic wounds shows early success in the treatment and acceleration of wound closure. Further investment within this area of treatment is likely to lead to a material which is able to positively affect chronic wounds, aiding in their closure, thus returning quality of life to patients affected.
